# “Well” off in animals: A taphonomic history of faunal resources and refuse from a well feature at Petsas House, Mycenae (Greece)

**DOI:** 10.1371/journal.pone.0280517

**Published:** 2023-03-01

**Authors:** Jacqueline S. Meier, Gypsy C. Price, Kim Shelton

**Affiliations:** 1 Department of Anthropology, Sociology and Social Work, University of North Florida, Jacksonville, Florida, United States of America; 2 SEARCH, Inc., New York, New York, United States of America; 3 Department of Ancient Greek and Roman Studies, University of California Berkeley, Berkeley, California, United States of America; Griffith University, AUSTRALIA

## Abstract

At the renowned archaeological site of Mycenae, striking depictions of animals in ancient art and architecture, such as the ‘Lion Gate’, reflect the great power of elite residents in the Late Bronze Age. To better understand how social complexity relates to human-animal interactions at Mycenae, more research is needed on the animals who actually lived there. In a first for the archaeological site of Mycenae, we utilized a contextual taphonomic approach and statistical analysis to study a faunal assemblage, focusing on a massive deposit recovered from a well feature located in Room Π of Petsas House. Petsas House was an industrial-residential complex at Mycenae used at least in part by ceramic artisans at the time of its destruction in the Late Helladic IIIA2 period. Intra-contextual analysis of the animal remains detected sub-assemblages with variable histories of animal use and deposition. The results revealed multiple disposal events and possible dog interments. Most of the refuse in the well likely originated from rubbish piles in the surrounding rooms and periphery that were cleaned after a destructive earthquake. Together, the faunal evidence yielded a more nuanced, possibly seasonal picture of animal access than previously available at this important political center. The results provide new insights into the diverse and resilient resource provisioning strategies available to extra-palatial residents of Mycenae, especially those who participated in craft production and trade networks at the height of the palatial period.

## Introduction

In the Late Bronze Age of Greece, animals were plentiful sources of subsistence and symbolism, as evidenced by preserved written records of mainly livestock, and striking images of animals in the art and architecture (e.g. the ‘Lion Gate’ at Mycenae). While ancient texts and artistic depictions reveal important details about past fauna, the biological remains of long-dead animals from Mycenaean archaeological contexts also preserve detailed information about animal management, use, and disposal in a variety of past social settings [1, 2]. Previous studies employed faunal taphonomic evidence in reconstructions of Mycenaean feasting and burial practices (e.g. [3–5]. More recent applications of taphonomic approaches explored the formation histories of faunal remains from a wider variety of Mycenaean contexts [6]. In this study, we utilize faunal taphonomic evidence to better characterize the animal economy and site use practices in an extra-palatial context at Mycenae.

Context-based taphonomic signatures of animal use and disposal have great potential to elucidate past differential access to animal resources among the residents of major political centers in the Aegean [7]. At the famous site of Mycenae in Greece, recent isotopic research of faunal remains revealed intra-site variation in the management of animals brought to the site in the Late Helladic (LH) IIIA2–IIIB period (1320–1190 BCE) [8]. As this suggests that many of the same types of animals differed in their biological histories, further research is needed to explore potential variation in the context-based histories of animal bodies at the site.

**Fig 1:**
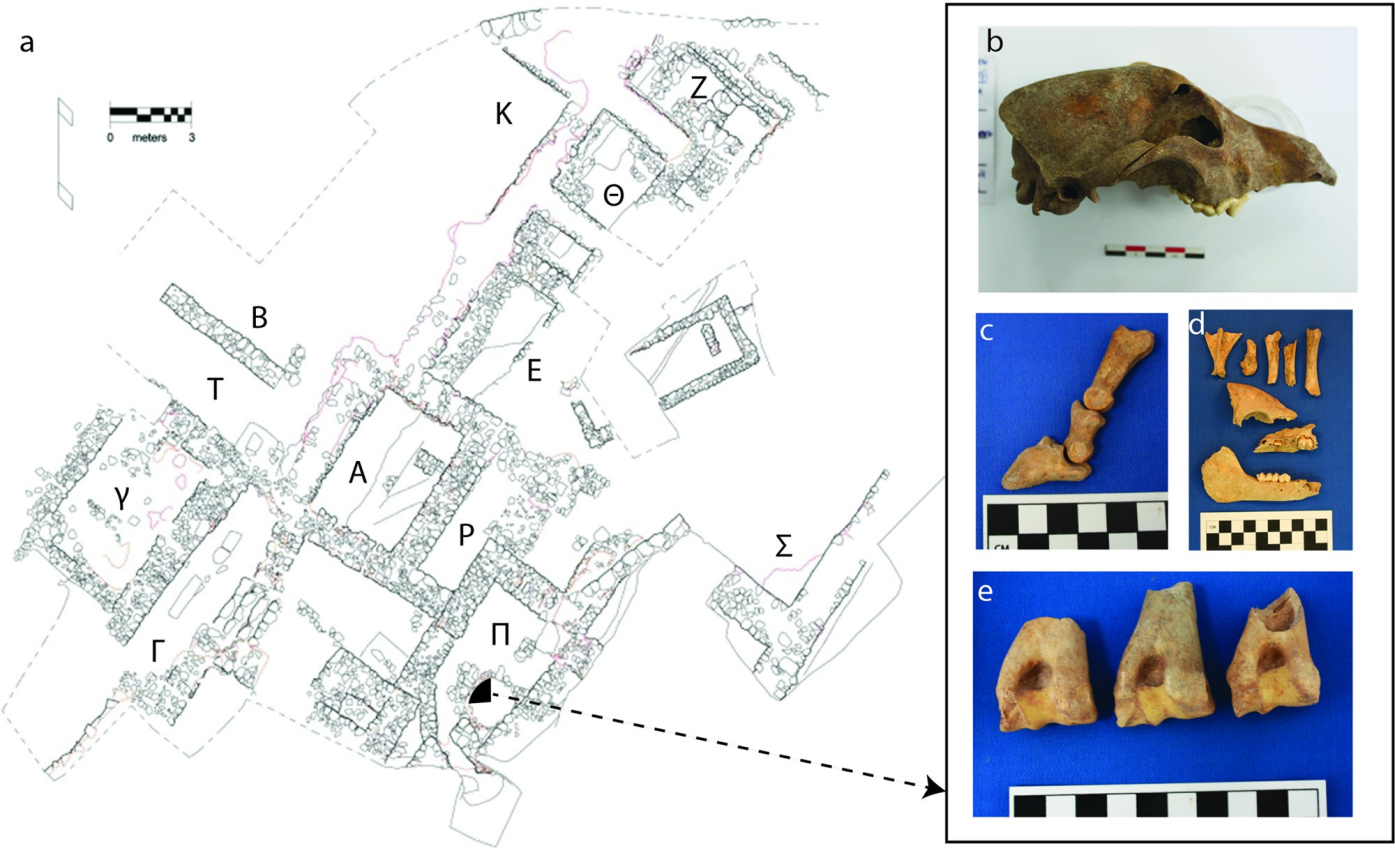
Map and examples of fauna from Petsas House, Mycenae. (a) Location of the well and northwest quarter sample area within the rooms of Petsas House (Greek letter labels) and examples of remains from the deposit that comprise (b) a dog skull, (c) a sheep foot, (d) a young pig skull and limb bones, and (e) several sheep humeri.

Our contextual taphonomic exploration of faunal remains recovered from Mycenae focused on Petsas House—an industrial and residential complex located within the settlement area (Fig 1a). Recent excavations of the complex by Shelton and her team for the Archaeological Society of Athens revealed that it likely functioned as an extra-palatial, craft-producing household in the LH IIIA2 period, or during the 14th century BCE (1390/70–1330/15 BCE [9]). Stacked vessels found in the house suggested that ceramic production and storage took place there, while the remains of cooking and food storage vessels reflected other, more domestic activities [10–12]. Animal remains were also recovered from different contexts within the complex, including an indoor well feature in Room П. Excavation of the well began in 2001, and by 2007, revealed a c. 12.35 meters deep deposit, densely filled with whole and fragmented ceramic, mudbrick, charcoal, glass, wall painting, stone, ivory, and other materials, including abundant faunal remains [11].

Typological study of the ceramic evidence from the well and rooms dated the deposit and the building’s destruction to a narrow timeframe during the later part of the LH III A2 period [13]. Calibrated radiocarbon dates were also obtained from two faunal bone samples from the well deposit. One specimen from level 93, one of the lowest levels excavated, dated to 1609–1415 cal. BC. Another was from a bone recovered near the middle of the deposit (level 42), dated to 1489–1235 cal. BC and this suggested a range that may exceed the standard dates for the LH IIIA2 period [14]. Some bone fragments, such as the former from level 93, possibly originated from disintegrated mudbrick destruction debris, some of which were found with ceramic sherd inclusions that date to the LH IIB and transitional LH IIB/IIIA1 period, around 75–80 years before the destruction event in the later LH IIIA2 period [11]. Still, these overlapping radiocarbon dates largely support the dates of the ceramic evidence and possibly suggest that more than one deposit of faunal remains, originally formed through earlier deposition events at different times in and around the house, were sourced for the refuse subsequently deposited in the well, even though the final deposition of the remains was within a narrow timespan overall. A faunal taphonomic analysis is needed to begin to reconstruct the depositional history of the well assemblage.

Diverse materials, including remains of animals (Fig 1b–1e) and destruction debris, were deposited in the well feature, filling it and much of the surrounding room [11]. Excavation observations of architectural destruction reflect an earthquake event. This is based on archaeological criteria established from other areas of Mycenae and other sites with evidence of similar natural disasters in the Aegean [15]. Notably, walls were uncovered that had shifted from their foundations and moved from their original courses [10, 11, 16]. There was abundant carbonized wood and burned materials likely from the damaged building structure found in the well deposit and in what was left of the rooms. These suggest a scenario involving localized fires, architectural destruction, and shortly after, the formation of the well deposit. During excavation and subsequent study, one of us (Shelton) observed that pottery sherds recovered from various layers of the well joined with vessel fragments found in several rooms of the building. This suggests that sherds and accompanying debris were likely moved from the rooms to the well. Given that the well was densely packed with material, and in much of the deposit, little soil was available to delineate a clear stratigraphic profile, we will use the faunal taphonomic evidence to explore potential intra-contextual variation in the types of refuse within the feature, in a similar way as the ceramic study. As this is the first study of its kind at Mycenae, the resulting taphonomic signatures of extra-palatial and domestic-industrial animals at Petsas House will inform future studies of refuse management and animal use in the wider settlement.

## Background

### Previous animal evidence from Mycenae

Early faunal research at Mycenae largely focused on symbolic representations, such as the iconic lions (e.g. on the Lion Gate and artifacts from Grave Circle A), and renderings of animals on ceramic vessels, wall paintings, and objects from the site, including a blue faience Monkey from Egypt [17–20]. These were used to support ideas about palatial power, access to rare resources [21, 22], and elite burial practices with canine companions [23–25]. Published zooarchaeological studies of Mycenae are relatively limited given the long history of excavations but include important work by Reese in the Southwest Quarter [26], Trantalidou in the Northwest Quarter [27], and personal communications shared by Albarella on the fauna from around the Cult Center area [28, 29].

The wider regional picture of animal use is informed by zooarchaeological studies of largely contemporaneous, Late Helladic sites in the Peloponnese, including Tiryns, Pylos, and Lerna, which indicate the prevalent economic use of caprines [5, 30, 31]. Other context-specific studies revealed more variation in animal use in the region, as cattle and pig remains were more abundant in some feasting and possibly sacrificial deposits and in some ritual-use areas [3, 5, 28, 32]. This variation is unsurprising given the exceptionally complex use of political, economic, and religious spaces at Late Helladic sites, including Mycenae [33].

### Recent faunal studies at Mycenae

Gypsy Price [8, 14] recently initiated a new era of analytical faunal research at Mycenae through a novel isotopic study of the animal remains from the Cult Center and Petsas House. Notably, Price detected dietary variation within and among animal groups from different site areas. At Petsas House, varying ranges of caprine isotopic values evidenced mixed herding strategies for sheep and goats managed in near and more distant locations [8]. Diets of pigs also varied between the Cult Center and Petsas House and may reflect a specific dietary regime used to alter the status of some pigs for ritual activities in the citadel. This suggests that a complex array of animal management goals provisioned different parts of the site.

The preliminary zooarchaeological study at Petsas House detected diverse taxa in a sample of the well deposit in Room П, including more abundant remains of pigs and smaller wild animals than most contemporaneous sites in the region [34]. The remains suggest reliable economic access to meat, including from dogs [35]. Still, the faunal economic record in the well may have been transformed by processes related to the earlier destruction event. Moreover, in the deepest levels of the feature, the excavators noted more highly abundant and complete dog remains than in the rest of the deposit [11]. This merits exploration of other potential faunal sub-assemblages within the well as they may be related to the use and deposition of different animals at the site.

## Methods

Bones, teeth, and shells preserve an array of pre- and post-depositional anthropogenic modifications (e.g. butchery marks) and natural damage (e.g. gnawing by carnivores or rodents). Owing to recent developments in the multivariate analysis of faunal taphonomic data [36, 37], improved reconstructions of depositional histories are achievable through refined, context-based comparisons [38]. Contextual taphonomic methods combine stratigraphic, zooarchaeological, and taphonomic data to explain intra-site and intra-context variability [39]. Faunal contextual taphonomy employs multiple scales of analysis to elucidate the past social activities and norms of refuse management involved in archaeological deposit formation (e.g. [40–43]).

This approach has great utility for studies of Mycenaean refuse management, as recently demonstrated by Macharidis at the site of Asine [6, 44]. Statistical correspondences between faunal specimens with different types of damage and different types of contexts (i.e. floors and weathered bones), reflecting faunal signatures of more and less cleaned refuse at Asine. At Petsas House, excavation observations of the destruction debris, ceramic evidence, and dog remains (above) inform our initial hypothesis that more than one type of refuse likely filled the well, and some cleaning activities were involved. We will test this hypothesis utilizing expectations of the faunal signatures of cleaning from Asine and explore other potential taphonomic evidence for Mycenaean depositional practice.

First, we analyzed multiple faunal taphonomic attributes represented across samples recovered from various depths in the well to detect different types of refuse. The potential refuse types include primary refuse deposited in the place of use with little subsequent movement, secondary refuse moved after initial deposition, such as by intentional cleaning or selective caching, and tertiary, or *de facto*, refuse that reflects a higher number of redeposition events, greater movement, and more exposure to post-depositional damage [45, 46]. More primary faunal refuse is often characterized by minimal bone movement, which includes evidence of exceptional preservation, high completeness of fragile bones, and, if intentional, more discrete deposits of specific taxa [38]. Conversely, more secondary or tertiary refuse should reflect more exposure to damage by natural agents (e.g. dogs, wind, water) and indirect human activities (e.g. trampling or construction disturbance), which largely obscure previously formed butchery marks and fresh bone breakage patterns [36].

We performed several tests to detect the type(s) of faunal refuse represented in the well. First, we explored potential variation in the evidence of minimal bone movement through tests of species diversity across samples of the assemblage by depth (following [38]). We also compared diversity index values for specimens identified to at least the genus level, excluding specimens identified to the body-size level (e.g. small bird) and indeterminate cervid and caprine specimens [47]. Chi-square tests of independence were employed to explore potential variation in the relative proportions of identified specimens of taxa across samples recovered from different depths (following [48]). Significant variation in the results was explored using adjusted residual values to compare deviations from expected values based on sample size (*α* = 0.05 unless otherwise stated).

Our analysis of the degree of post-depositional movement compared the quality of bone preservation across samples from different levels. Tests of variation in the preservation of different types of damage utilized chi-square tests of independence of the relative proportions of different taphonomic attributes (%NISP), represented in the sample of medium-sized ungulate bone specimens (caprine, pig, and medium ungulate body-size categories). This minimized the potential for variation in damage due to the variable surface areas and structural densities of bones of differently sized animals [49].

Next, we made clusters of several adjacent level group assemblages based on the taxonomic and taphonomic attribute abundances (%NISP, cluster analysis with stratigraphic constraints performed in PAST, [50]). The resulting sub-group samples were used to explore potential variation in age and body part groups within the abundant pig and caprine samples. The aging analysis utilized established rates of bone fusion [51–53], rather than tooth eruption and wear, as this allowed for a more direct comparison to the medium-sized ungulate bone preservation results. Intra-contextual body-part representation analysis compared percentages of the minimum number of anatomical units (%MAU; following Stiner [54]). The pig and caprine results were compared to those of dogs as these animals often have different social relationships with humans that impact their use and deposition. Finally, tests of density-mediated attrition utilized caprine element portion survivorship and goat limb bone photon densitometry values [55].

### Data collection

Data collection took place in Greece at the Archaeological Museum at Mycenae. To capture a representative taphonomic picture of the massive well deposit, we analyzed a vertical sample of faunal remains from all level units that included the northwestern quadrant. The excavation strategy for each level depended on the circumference of the well feature and the volume of material at each given depth. Levels were excavated in 10 cm spits, or arbitrary levels, and subdivided into smaller units within, denoted by cardinal direction due to the varying artifact densities, up to 1/2 standard Greek excavation basket volume (for example: [56]). Thus, the vertical samples are unequal in their total representation of the excavation layer volume across the width of the well feature. The estimated percentage of each level represented by each excavation sample is listed in S1 File. Samples spanned level 5B, where the well was first defined as a feature, to level 98, where excavation ended at a large stone block, likely a fragment of the wellhead that fell in after the earthquake. Thus, the lowest deposits of material in the well are on top of the block and post-date the destructive event that likely caused the block to fall in. Excavation of the well ceased at the block, as it was too dangerous to remove from a confined space over twelve meters below the surface.

Zooarchaeological and taphonomic data were collected from a total sample of 13,322 identifiable faunal specimens. Specimens were identified to the species, body part, the portion of the element, and age when possible. Data were recorded on the stages of weathering, using Behrensmeyer’s scale (not weathered [stage 0], weathered [stages 1–2 = low degree, stages 3–5 = high degree]), burning (Stiner’s scale: not burned [stage 0], burned [stage 1–3 = carbonized, stage 4–6 = carbonized]), types of bone breakage (green, dry, or excavation breaks), and the presence of cut and gnaw marks [57–59].

**Fig 2:**
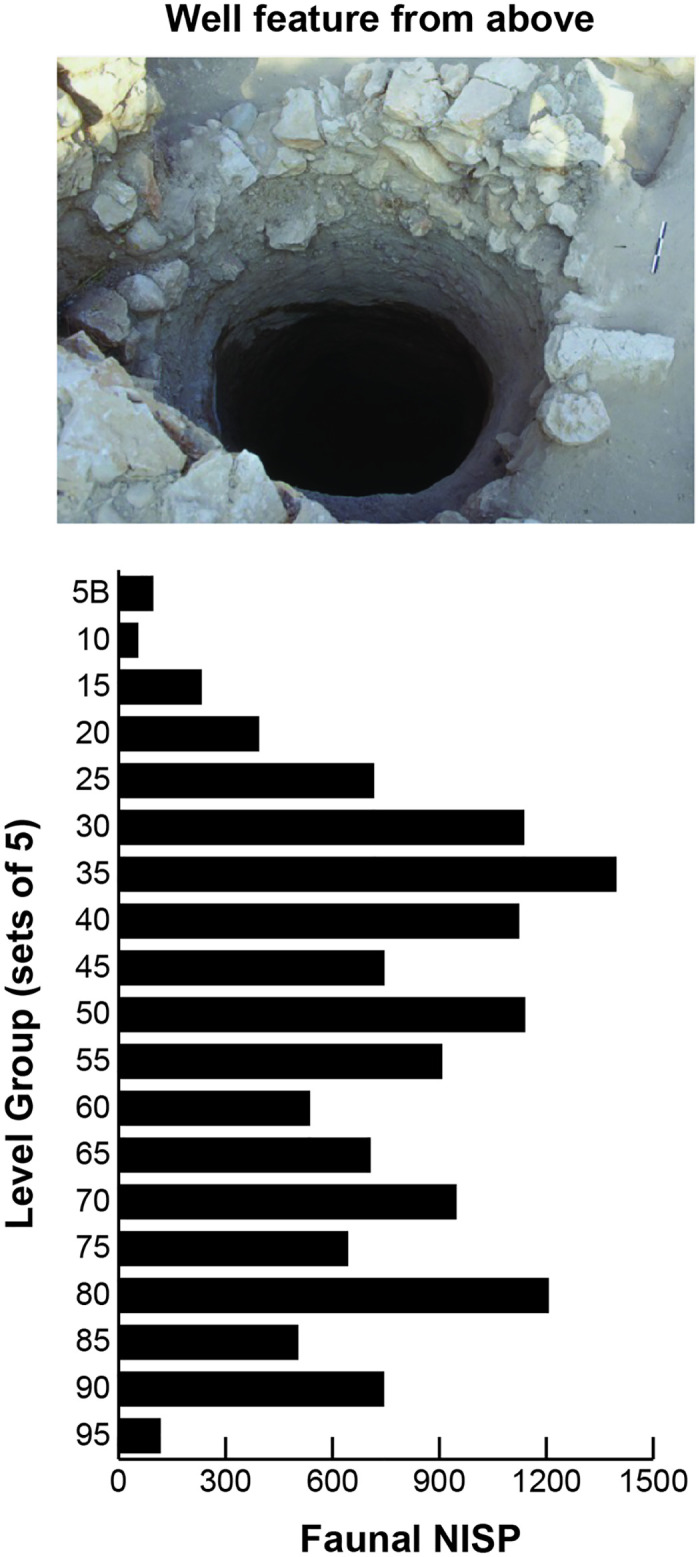
Samples sizes of identifiable* faunal remains from the well (pictured). *Includes specimens identified to more and less general animal categories.

Given the absence of visible stratigraphic divisions in sediment, sub-assemblage samples of levels were grouped by increments of five. Hereafter these arbitrary samples are termed the “level groups”, designated by the level number in the group that was closest to the surface (e.g. level group ‘15’ represents the combined five samples recovered from levels 15–19). The formation of level groups maximized the sample sizes for statistical analysis. Still, some sample sizes remained small, especially in the uppermost (5 and 10) and lowest level groups (95) (Fig 2, S1 File).

## Results

### Taxonomic abundances

**Fig 3:**
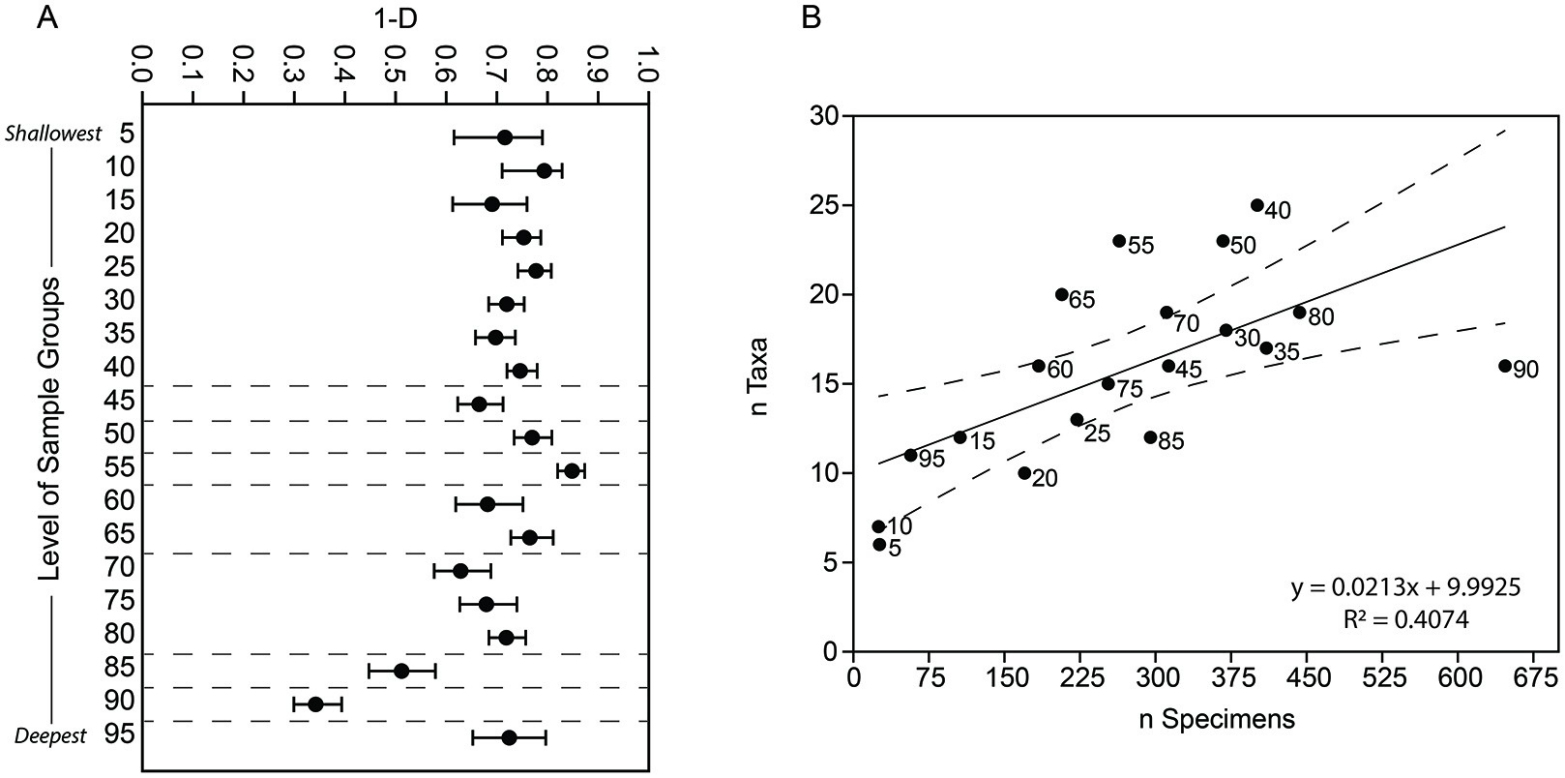
Comparisons of the species diversity of each sample (level group) by depth based on (A) Simpson’s index values and (B) numbers of taxa and specimens. Dashed lines highlight the significant differences in adjacent samples for A and the 95% confidence interval for B. Includes only specimens identified at least to the genus level.

Of the identified faunal remains from the entire well sample, pigs were the most highly represented species (40% of the total NISP), followed by caprines (29%), cattle and dogs (both 8%), and many wild species (Table 1). Across the well samples, diversity indices varied significantly, especially in the lower half of the deposit (Simpson’s index 1-*D*, Fig 3, S2 File). Diversity was highest for level group 55 and lowest for 85 and 90. Still, a modeled linear bivariate regression of taxonomic richness and sample size detected a weakly significant correlation, suggesting that changes in the number of taxa across the level groups may not be meaningful (least squares regression: y = 0.0213x + 9.9925, r^2^ = 0.40, p = 0.0032).

**Table 1:** Relative abundances of species within the sample from the well deposit (detailed information in S1 File).

Taxon	NISP	%NISP
*Pig*	4871	41.1%
*Caprine*	3501	29.5%
*Cattle*	940	7.9%
*Dog*	918	7.7%
*Equid*	6	0.05%
*Cervid*	94	0.7%
*Wild Carnivore*	66	0.5%
*Hare*	401	3.3%
*Bird*	297	2.5%
*Fish*	241	2.0%
*Rodents/Insectivores*	22	0.1%
*Shell*	702	5.9%
*Total* *	11,858	100%

*General categories of medium-sized ungulate remains excluded from Total and %NISP calculations (n = 1,464)

**Fig 4:**
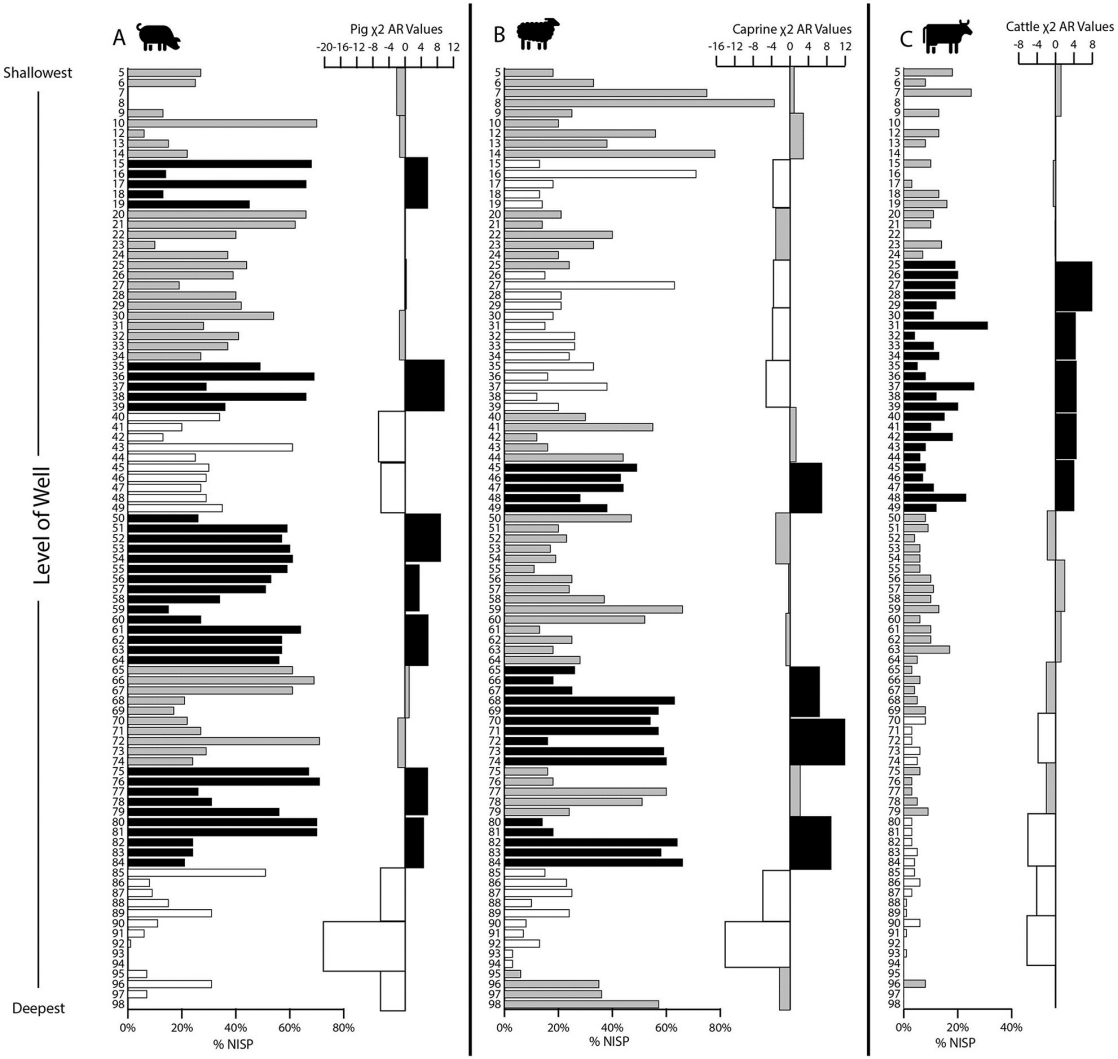
Species abundance (%NISP by level) and χ^2^ adjusted residual values by level groups for livestock (a) pig, (b) caprine, and (c) cattle remains. Colors reflect significance of *p* ≤ 0.01 for *df* = 198. Adjusted residual values of level group samples with significantly higher proportions (≥ 3.2; *black*), significantly lower proportions (≤ -3.2; *white*), and no significant variance or n < 5 (*grey*). Note: Sample levels were arbitrary 10 cm spits or strata, not cultural or soil levels, and excavated lots within the levels were based on material volume (filled excavation baskets).

Further chi-square tests indicated significant variation in relative species abundance across the level group samples (%NISP; Pearson’s χ^2^ = 13433.38, *p* < 0.001, *df* = 216, φ_c_ = 0.279; S3 File). Given the wide range of sample sizes and variables, a higher threshold of significance was required (1%; adjusted residual values ≥ 3.2 and ≤ -3.2). Significantly higher proportions of pig remains were detected for many level groups, except for samples from the lowest part of the well (adjusted residual values ≥ 3.2 in level groups 15, 35, 75–80; Fig 4a). By contrast, there were higher proportions of caprine remains in level groups from the middle (45) and lower parts of the deposit (65–70, 80; Fig 4b). Interestingly, one block of level groups in the mid-to-upper part of the deposit contained a significantly higher proportion of cattle remains (25–45; Fig 4c). Remains of deer were also more highly represented in the middle of the deposit (35, 50; in S3 File).

**Fig 5:**
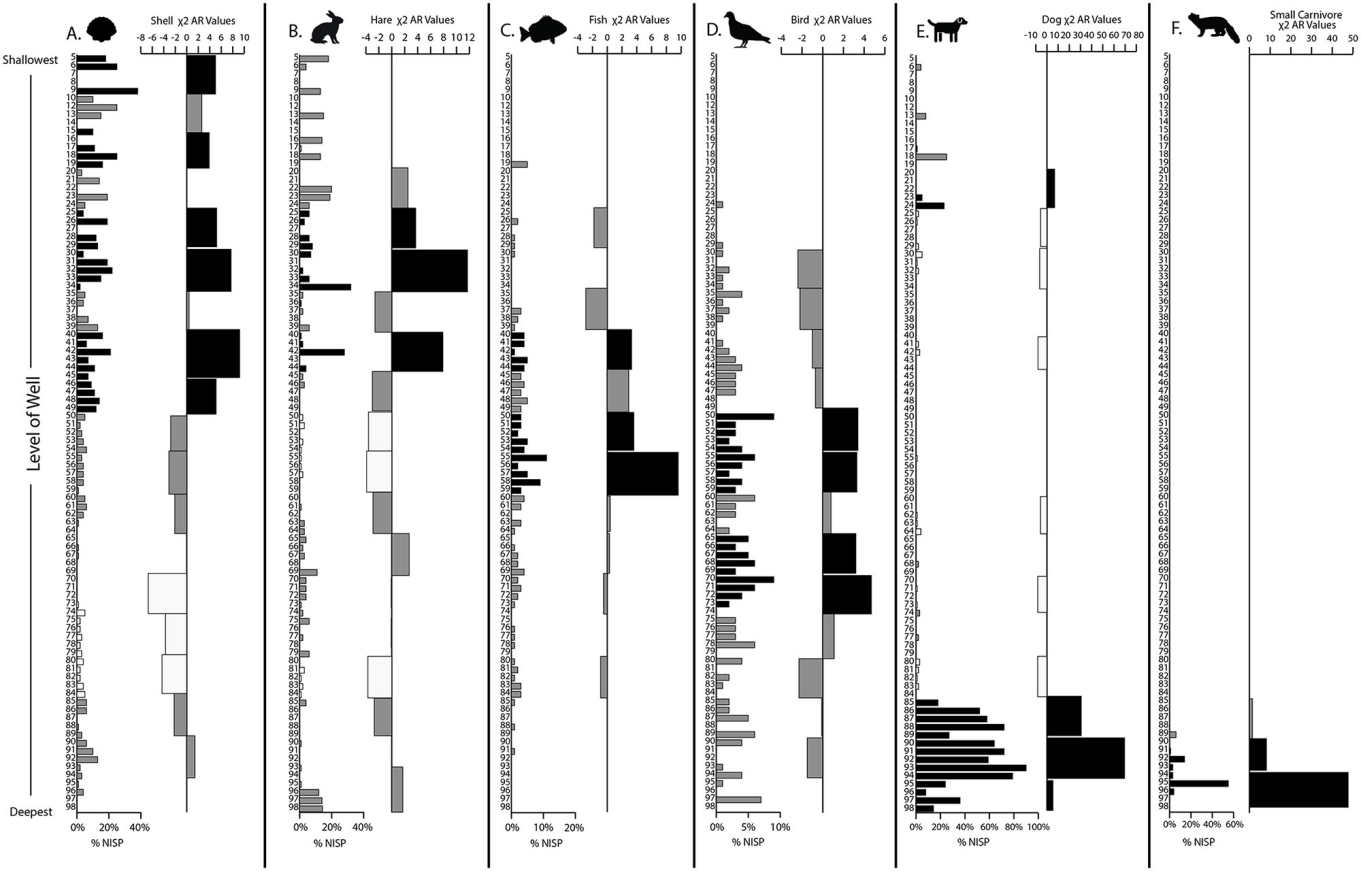
Relative species abundances by level (%NISP) and level group with χ^2^ adjusted residuals plotted for (a) shell, (b) hare, (c) fish, (d) bird, and (e) small carnivore remains (see Fig 4 legend).

The same chi-square test indicated significant shifts in the abundances of smaller taxa remains by depth (Fig 5a–5f). The bottom of the well comprised higher proportions of carnivore remains, mostly of martens (level groups 95–90), and dogs (95–85) (Fig 5e and 5f). Several middle deposit samples contained higher proportions of bird (70–65 and 55–50) and fish remains (55–50 and 40) (Fig 5c and 5d). Finally, some upper deposit samples had more hare (45 and 35–30; Fig 5b), shell (45–40, 30–35, 15, and 5; Fig 5a), and dog remains (20).

### Damage and preservation

Evidence of bone damage was limited overall (Table 2). The most prevalent type of damage was due to weathering and most bones reflected only a low degree of weathering damage. Gnawing by carnivores and rodents had little impact. Evidence of the preservation of fresh bone damage included some butchery marks. For limb and foot bone elements, evidence of green bone breakage was more abundant than dry bone breakage and completeness was high, indicating limited post-depositional destruction.

**Table 2:** Summary of taphonomic damage for the entire faunal assemblage.

Type of Damage	NISP	%NISP
*Total Weathered*	1235	9.3%
*High-degree weathered*	80	0.6%
*Low-degree weathered*	1155	8.6%
*Gnawed*	180	1.3%
*Complete elements*	2799	21.0%
*Butchery marked*	312	2.3%
*Total Burned*	126	1.0%
*Burned white*	49	0.4%
*Burned Black*	77	0.6%
*Green bone breakage* *	1653	76.0%
*Dry bone breakage* *	527	24.0%

*Breakage types exclude new excavation breaks and only include limb and foot bone elements

**Fig 6:**
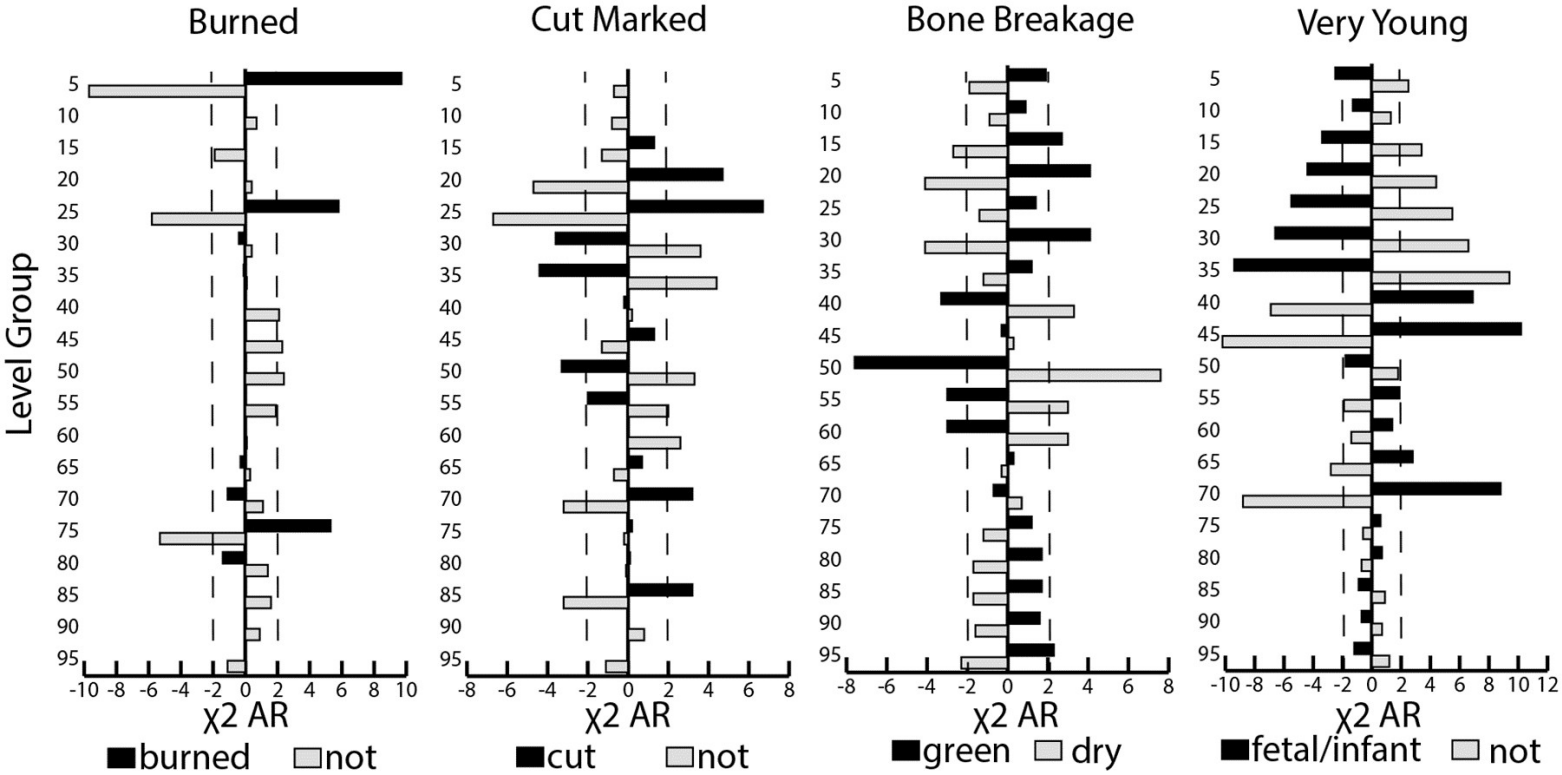
Plotted χ^2^ adjusted residual values for medium-sized ungulate bones with surface damage across level groups (NISP). The significant adjusted residuals extend beyond the dotted line in either direction (≤ -2, ≥2).

Tests of significant differences in medium-sized ungulate bones with taphonomic damage indicated variation among the well samples (Fig 6). Upper and lower deposit samples contained significantly higher proportions of burned (level groups 5, 25, and 75) and cut-marked bones (20–25, 70, and 85) than samples from the middle deposits (burned vs. not burned χ^2^ = 180.852, *df* = 18, *p* < 0.0001, φ_c_ = 0.143, S4 File; cut vs. not cut χ^2^ = 139.511, *df* = 18, *p* < 0.0001, φ_c_ = 0.125, S5 File). This was also true of the green bone breakage evidence, much of which likely reflects fresh bone processing for food. Green breakage was more highly represented in several upper and lower level groups (15, 20, 30, and 95; green vs. dry breaks χ^2^ = 140.609, *df* = 18, *p* < 0.0001, φ_c_ = 0.150, S6 File). By contrast, the middle deposit samples contained significantly higher proportions of fragile bones of very young (fetal or infant) specimens of pigs and caprines (70–65 and 45–40), reflecting exceptional preservation (very young vs. not χ^2^ = 417.173, *df* = 18, *p* < 0.0001, φ_c_ = 0.208, S7 File). Given that smaller, more tender carcasses of young animals often require less processing effort and provide only limited yields of food for intensive butchery, this may explain why the prevalence of cut marks and green breakage is significantly lower for samples with more remains of young livestock. Also, more fragile young animal remains were likely more susceptible to post-depositional breakage, which would effectively erase much evidence for green bone breakage.

**Fig 7:**
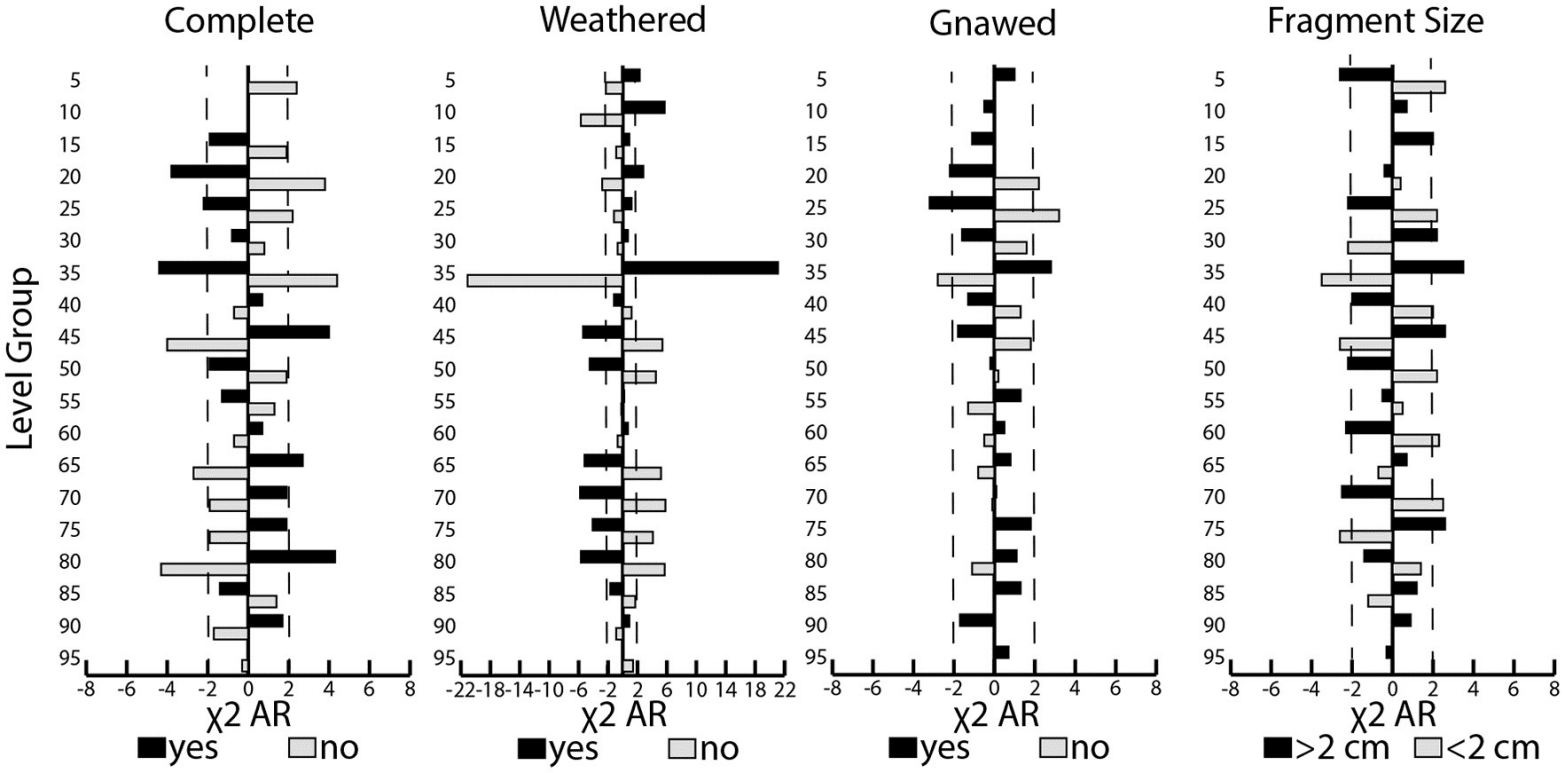
Plotted χ^2^ adjusted residual values for tests of medium-sized ungulate bones with other types of damage across level groups (NISP). The significant adjusted residuals extend beyond the dotted line (≤ -2, ≥2).

The other evidence of damage to medium-sized ungulate bones varied by depth (Fig 7). We detected a significantly higher proportion of complete than incomplete bones in several samples (level groups 45, 80, and 90), reflecting less damage and better preservation in the middle and lower parts of the deposit (complete vs. not χ^2^ = 101.522, *df* = 18, *p* < 0.0001, φ_c_ = 0.107, S8 File). Also, the upper deposit samples were exposed to significantly more weathering (level groups 5, 10, 20 & 35; weathered vs. not weathered χ^2^ = 594.119, *df* = 18, *p* <0.0001, φ_c_ = 0.259, S9 File). One upper deposit sample may have been more gnawed (35), but the strength of association for this test was weak (gnawed vs. not χ^2^ = 42.208, *df* = 18, *p* = 0.001, φ_c_ = 0.069, S10 File). Samples with significantly higher proportions of larger (>2 cm) than smaller (<2 cm) bone fragments without new breakage were mainly detected in the upper half of the deposit and the strength of this association was also weak (15, 30–35, 45, and 75; larger vs. smaller fragments χ^2^ = 67.548, *df* = 18, *p* <0.0001, φ_c_ = 0.087, S11 File). Together, this indicates that the samples from the upper deposit were more exposed to post-depositional damage. The potential size sorting also may reflect how some parts of the deposit were more altered by movement prior to final deposition, such as through cleaning activities (e.g. [38, 60]). Post-depositional size sorting after deposition in the well, while possible, is unlikely given the lack of sediment and space for bones to move through in the feature.

### Taphonomic sub-assemblage clusters

**Fig 8:**
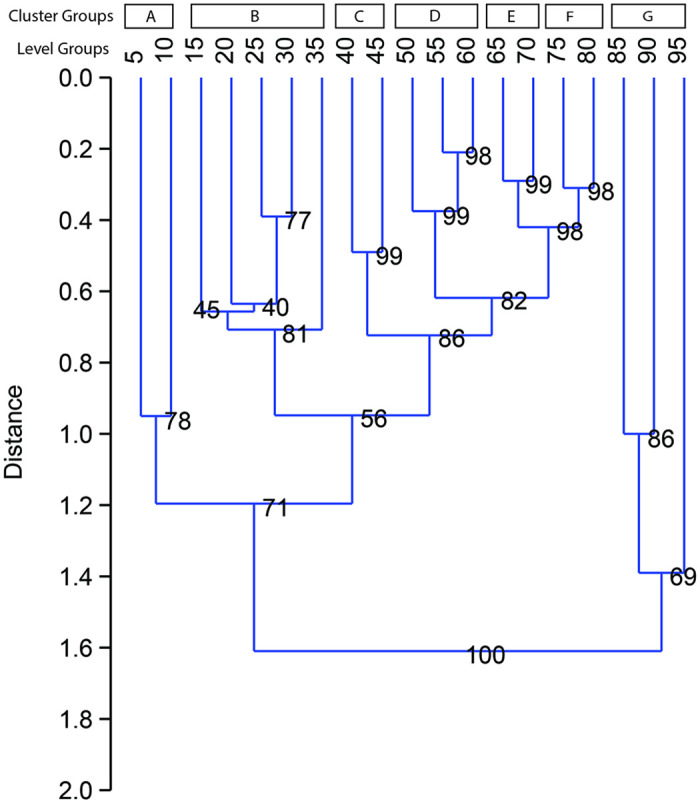
Output of cluster analysis (produced in PAST and stratigraphically constrained with CI%).

The results highlighted variation in the faunal attributes across the deposit. Next, we employed hierarchical cluster analysis with stratigraphic constraints to combine the results and explore the relative distances between adjacent samples (Manhattan similarity index of %NISP values of species and taphonomic attributes, bootstrapped n = 999). This exploratory analysis formed two main clades (Fig 8). One exhibited a high degree of distance among the samples from level groups 85–95. The other consisted of one secondary cluster of level groups 5–10 and another of less distantly clustered level groups 15–35, 40–45, 50–60, 65–70, and 75–80. These cluster groupings were given letter designations for subsequent analyses of intra-contextual variation. Although they clustered more distantly, the lowest level groups of the same clade (85–95) were grouped (cluster group G) based on the estimated separation from the main clade.

**Fig 9:**
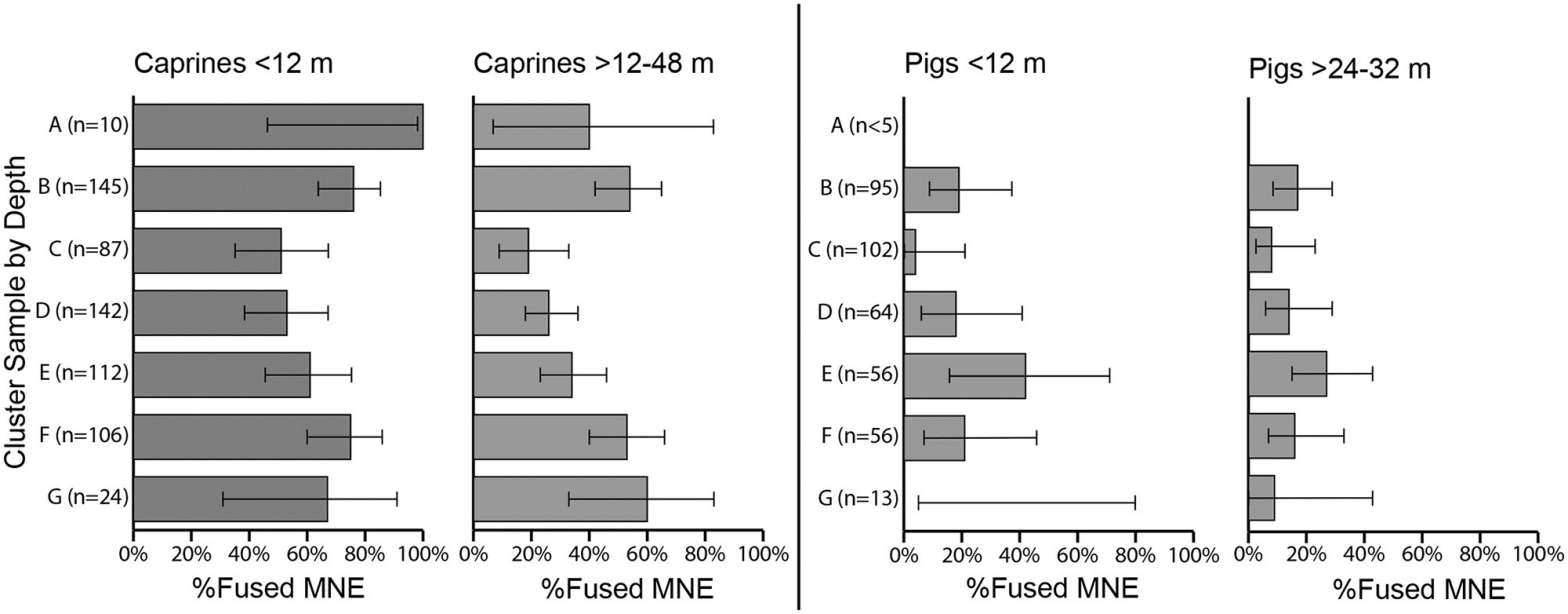
Age analysis of survivorship (%Fused Bone MNE) for caprines and pigs at young and older age stages by level cluster groups with confidence intervals (Wald test). Note: n < 30 for Clusters A and G.

Among the cluster samples, there was some variation in caprine and pig survivorship (%Fused MNE; Fig 9, S12 File). Survivorship up to one year of age was lower for the caprines represented in clusters C and D and higher in cluster B (χ^2^ of MNE = 15.08, *df* = 6, *p* = 0.0196, φ_c_ = 0.24). Of the caprines that lived beyond one year of age, survivorship fluctuated significantly by depth from a high level in cluster F to less than a quarter of the population in cluster C, and a higher level again in cluster B (χ^2^ = 25.868, *df* = 6, *p* = 0.0002, φ_c_ = 0.28). For the pigs, survivorship was low overall, especially for the sample in cluster C, and variation among the samples was not significant (Fused and Unfused MNE of elements up to 12 months of age χ^2^ = 8.425. p = 0.0606; Fused and Unfused MNE of elements beyond 24 months of age χ^2^ = 8.460, *p* = 0.1326).

By comparison, tests of the only sizable bone fusion aging samples for dogs, from clusters B (n = 28) and G (n = 140), revealed high levels of survivorship overall. In the lower deposit (cluster G), nearly all dogs represented lived to at least 12–18 months of age (97% fused MNE), and at least one dog died before six months of age. In the upper part of the deposit (cluster B), most of the dogs represented likely reached at least 10 months of age (95% fused) while slightly fewer lived to 12–18 months of age or older (71% fused). Significant variation was only detected among the cluster samples in the older age range; the dog remains of Cluster G reflected higher survivorship beyond one year of age than those of Cluster B (S12 File).

**Fig 10:**
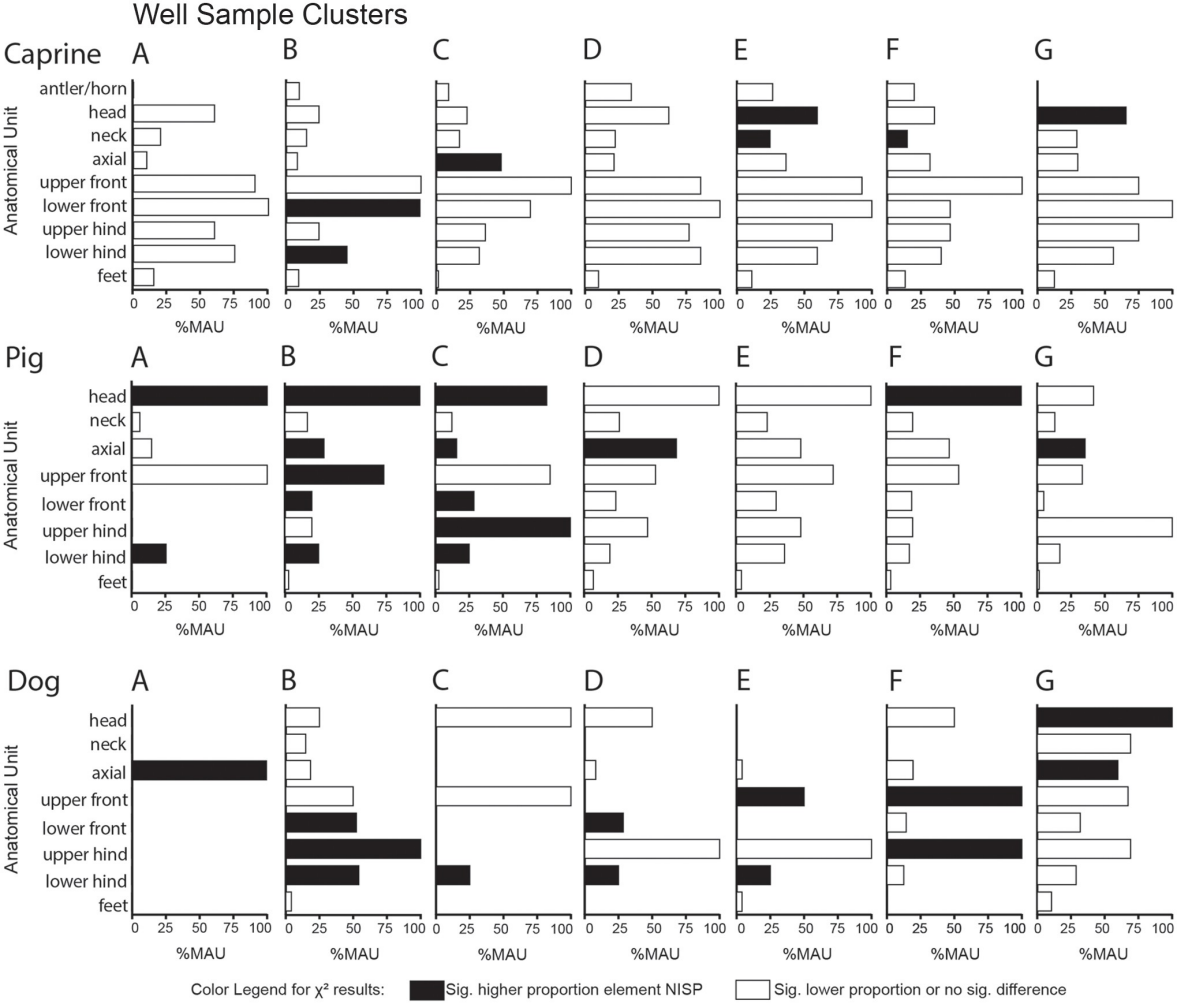
Caprine, pig, and dog body-part representation by sub-assemblage cluster (A-G). Anatomical units with significantly higher NISP proportions per element are marked in black (*p* > 0.1, S13–S15 Files).

Body-part representation also varied significantly across the clustered samples (Fig 10, S13–S15 Files). For caprines, nearly all anatomical units were highly represented, especially the limb parts. Further tests indicated significantly higher proportions of the head, neck, and axial element fragments (%NISP) in the lower samples (indicated with black shading of the corresponding anatomical unit of the MAU bar chart). There were higher proportions of caprine limb element specimens (radius, tibia, and calcaneus) in cluster B (χ^2^ Element NISP/cluster = 464.69, p = 7.3283E-44, *df* = 144, φ_c_ = 0.155). Pig head parts were more highly represented as were several pig limb elements, particularly in clusters B and C (χ^2^ Element NISP per cluster = 608.88, *df* = 120, p = 1.83E-66, φ_c_ = 0.153). By contrast, dog body parts were more evenly represented and reflected higher carcass completeness in the samples with more abundant dog remains (B and G; see NISP values in S15 File). Tests of density-mediated attrition of the caprine remains found no significant correlations (S16 File).

## Discussion

The abundant faunal evidence made possible the first detailed taphonomic reconstruction of animal use and deposition at the height of the palatial period of Mycenae, during the LH IIIA2 period. The results revealed nuanced variation within the faunal assemblage, highlighting how several different types of refuse came to fill the feature. The findings provide new insights into the treatment of dogs, cleaning practices, and food provisioning strategies of a Mycenaean household.

### The depositional history of the well

Beginning at the bottom of the deposit, (Cluster G) stands apart, comprising mainly carnivore remains. In the lowest excavated level group, 95, martin remains and a few medium-sized ungulate bones with green bone breaks indicate limited post-depositional damage. Above this (level group 90), there were highly complete remains of mainly dogs and a few of rodents. Further above, level group 85 was also dominated by dog remains, but less so, and contained more cut and incomplete remains of medium-sized ungulates likely reflecting processed food refuse. This suggests that bodies of dogs, martens, and rodents were covered over in the well by the refuse of meals, possibly in associated or closely timed events. In all, the evidence of minimal bone movement after deposition and excellent preservation reflects mainly primary refuse at the bottom of the well (Table 3).

**Table 3:** Summary comparison of results across clusters of level groups from the well.

Cluster	Max Dist	More abundant taxa	Burned	Cut	Weath-ered	Gnawed	Complete	Green Breaks	Bigger >2 cm	Very young	Caprine Results*	Pig Results*	Refuse type
*A (5–10)*	.3	Shell	+		+		-		-	-	n/a	Head, Limb	3º
*B (15–35)*	.3	Pig, cow, deer, dog, hare, shell	+	+/-	+	+/-	-	+	+/-	-	Older, Limb	Head, Limb	3º
*C (40–45)*	.18	Caprine, cow, hare, fish, shell			-		+	-	+	+	Younger, Axial	Head, Axial & Limb	2º
*D (50–60)*	.09	Pig, deer, fish, bird		-	-		-	-	-		Younger	Axial	2-3º
*E (65–70)*	.08	Caprine, bird		+	-				-	+	Head, Axial		2º
*F (75–80)*	.08	Pig, caprine	+		-		+		+		Older, Axial	Head	2º
*G (85–95)*	.55	Dog, marten		+			+/-	+			Head	Axial	1º

Note: + = sig. higher proportion, - = sig. lower proportion, blank = no sig. variation from expected proportion.

*Only results of body part and age attributes with significantly higher proportions listed.

The samples above, clusters D-F, were more diverse in terms of taxa and contained increasingly pronounced post-depositional damage and younger caprines nearer to the surface as the well was filled. In the lowest sample, cluster F, more large fragments reflect some movement of more impeding refuse to the well. Cluster E was well preserved with more remains of very young livestock, unweathered bone, and more cut remains processed for food. Above, cluster D reflected more post-depositional destruction overall but also some fragile head parts. Together, the mid-to-lower cluster samples were slightly more damaged and thus were likely deposits of moved refuse. This indicates that more secondary, and possibly tertiary, refuse accumulated in the middle of the well feature, possibly during a large-scale cleaning event.

Further above, the upper-middle deposit samples differ markedly in form, especially in cluster C, and notably contained more shell, hare, fish, and cattle remains. In cluster C, the youngest survivorship levels were detected for the caprine and pig remains, indicating excellent preservation of fragile bone. More large, complete fragments with dry breakage were present and likely indicate size sorting from cleaning and limited food processing. The considerable dry bone breakage also suggests damage by post-depositional movement, yet the high level of body part completeness also suggests formation by a limited number of deposit events.

The upper part of the deposit contained more distantly clustered faunal samples. Cluster B contained remains of diverse taxa with more surface damage, likely reflecting food refuse with more post-depositional exposure. Level groups within this cluster also shifted from more large, gnawed fragments to smaller, less damaged fragments above. The size sorting may indicate a sequence of events where the more exposed refuse from the tops of rubbish piles was first cleaned away and moved to the well, followed by the more intensive clearing of smaller, less-exposed fragments left behind in the first pass of cleaning (see [60]). Together, the evidence for greater movement points to more tertiary refuse in cluster B.

Even greater movement of refuse was evidenced at the top of the deposit sample in Cluster A. More weathered and burned remains of smaller, less complete fragments reflect the highest degree of post-depositional damage in the well deposit. The uppermost samples also contained more remains of shells, mostly of the *Arca noa* variety, than the rest of the well deposit.

The faunal taphonomic patterns reveal a sequence of depositional events, marked by better preservation in the deeper samples and greater movement and damage of refuse nearer to the surface. As the well was filled, refuse types shifted with some overlap likely due to the sloping of the deposits noted during excavation. While we do not attempt to draw fine lines between individual levels with the faunal data alone, as it was deposited along with over 10,000 ceramic vases, over 100,000 ceramic sherds, and other finds, the taphonomic results support the following provisional depositional history for the faunal remains:

1. After a large piece of the wellhead fell into the feature, several martens fell in or were deposited on top.
2. Highly complete dog carcasses came to be deposited next, along with some food refuse and rodents.
3. Next, there was deposition of mainly pig and caprine remains that were not highly processed and were somewhat burned.
4. Afterwards, remains of mainly young livestock animals and more small wild game were cleaned and moved to the well from midden deposits elsewhere.
5. Finally, more disturbed refuse deposits were moved to the well, and refuse continued to accumulate for another meter above the feature context, spilling into the surrounding room.

### Are there feasting or mortuary deposits of animal remains in the well?

The deepest part of the well (cluster G) contained more primary faunal refuse than the rest of the assemblage. The evidence for excellent preservation and high completeness likely supports a scenario of rapid disposal that ultimately protected the dog and marten bodies as well as some inclusions of rodents and food refuse. At the bottom, there was little evidence of the impact of deposition from several meters above or the crushing load of subsequent deposits of heavy materials and debris. This may indicate that the carnivore carcasses were largely intact, and possibly fleshed bodies when deposited. It may also point to some water in the well at the time of deposition, although this would likely also have led to more disarticulation, polishing, and movement of more buoyant elements that is similar to cave deposits with water [61]. Still, there was ceramic evidence of more fresh breaks in the shallower levels, about four meters from the surface, than in the deeper parts of the deposit that may relate to water in some parts of the well.

Why the carnivore carcasses ended up in the well is less certain. The surrounding bone remains do not reflect gnawing, which could be expected if the martens and dogs fell in alive and tried to survive for long afterwards. While there is no evidence that the martens were intentionally dispatched or used before disposal in the well, the dogs may have been killed by people or during the destruction event. One faint, possible cut mark on a dog cervical vertebra was noted in the deepest deposit layers from the wider well assemblage beyond the northwest quarter sample featured in this study. This may suggest that at least one dog was possibly dispatched with an implement before deposition in the well.

Furthermore, the association of dog, marten, and rodent remains appears to reflect the convenient disposal of commensal animals in the well, rather than evidence of a special location for ritual animal deposition. Still, dog remains have been frequently recovered from more sacred types of ritual contexts at Mycenae, including Chamber Tombs 505 (LH IIIA2) and 533 (LH IIIA-B) ([21] *in* [62]). Dog remains were also recovered from a well feature near the Cyclopean Terrace Building at Mycenae along with the remains of three people ([63], p273). The placement of dog bodies in wells may represent a less remarkable type of intentional mortuary treatment related to the deposition of dogs in the tombs. Thus, dogs may have been afforded a more substantial degree of personhood in death than other types of animals at Mycenae. Further taphonomic research is needed to compare the dog remains from different contexts.

To better situate the dog remains within the depositional history of the well, we must consider the surrounding faunal refuse. Above Cluster G (with the highly complete dogs), Cluster F included more complete and burned remains of caprines and pigs than other clusters, which are often seen as indicators of ritual animal use at contemporary sites. While this evidence stands apart, the percentage of burning is low overall and represents mostly carbonized, not calcined bone. The highest degree of burning found in the well was still quite minimal (3% NISP burned in level group 75). Additionally, the burning impacted diverse remains of hare, cattle, and shellfish, rather than those of a specific taxon. This appears to represent more indiscriminate and indirect thermal alteration more akin to damage caused by a fire near a refuse deposit [e.g. [58]] than the prescribed actions of a ritual practice.

Based on wider regional evidence, directly burned, sacrificed carcasses would more likely target specific animals or parts and lead to more calcined (white) bone remains. This includes the ritual evidence from Pylos, where several discrete deposits of mostly whole cattle bones from a ceremonial area were more ubiquitously burned (81–100% burned MNE) and interpreted as evidence of burned sacrifices [64]. Also, at Ayios Konstantinos at Methena, burned bones of mostly young pigs and other animals from the hearth area of a sanctuary complex were interpreted as remains of burnt animal sacrifices and possible feasting activities [28]. Similarly, burned young pig bones recovered from a drain of Megaron B at Eleusis were interpreted as a burned animal sacrifice [65]. The outlier of the comparative sample is from Tsoungiza, where the animal remains associated with feasts did not evidence overtly special selection or treatment, even though the wider ceremonial context and paraphernalia appeared to support the interpretation of the faunal remains as a component of the feasting refuse [3].

The faunal evidence from the Petsas House Room Π well differs from wider regional examples of special rituals or offerings involving fire and animals. Further tests of whether the faunal refuse from the well came from special animal consumption events will require knowledge of the associated materials. Based on the faunal information alone, the secondary refuse above the dog deposits likely reflects the use of the well as a repository for cleaned domestic rubbish that was slightly burned by accidental exposure to heat, likely during the destruction event or by earlier haphazard disposal near hearths or kilns.

### Does the faunal evidence reflect cleaning activities?

In the well, the refuse of meaty meals and other activities with animals was differentially preserved by depth and appeared to reflect much pre- and post-depositional damage caused by cleaning activities. The rubbish likely originated in areas with varying degrees of active use, reflecting a cultural norm of cleanliness that tolerated fairly high amounts of refuse in living spaces. The variation in refuse types also appears to reflect the prior exposure of remains that were more and less completely buried within household rubbish heaps before they were moved to the well. Given that the great density of material packed into the well deposit would likely have minimized much further movement, the size sorting evidence suggests a cleaning sequence that first relocated the larger fragments from a rubbish pile to the well, then returned to gather up the smaller fragments left behind, repeating this process to fill much of the middle of the deposit. Overall, the heterogeneous composition and treatment of the cleaned refuse likely represents a longer period of diverse consumption events than would be expected for a feast or a single repeated type of ceremonial trash deposition.

Although the deposition of refuse in the well feature led to the excellent preservation of much of the assemblage, bones from the upper deposit were less protected from post-depositional damage. Here, remains were more weathered but to a low degree [57]. Much of this damage was likely caused by longer exposure times before the final deposition or burial in the well. This is supported by the wider evidence for gnawing and dry bone breakage in samples near the surface. Still, the remains may also have been weathered after deposition in the well by fluctuations of wet-dry cycles known to create similar low-grade weathering damage [66]. Future examination of the faunal remains recovered from deposits in the surrounding room above will investigate these post-depositional processes.

More broadly, the combined evidence of burning, weathering, and gnawing damage in the upper part of the deposit appears similar in form to the faunal samples from several floor deposits at Middle-Late Helladic Asine [44]. The similarities confirm that faunal remains exposed to diverse and substantial damage likely represent more mundane refuse. This taphonomic signature provides a useful baseline to better define household refuse from ceremonial trash, as interpretations of the latter are abundant in Mycenaean faunal studies. Our results also support a new hypothesis. Even though some cleaning of material from the house to the well likely occurred after the destructive earthquake at Petsas House, a low level of cleanliness appears to have been the norm for the everyday use of mundane household spaces during the latter part of the LH IIIA2 period. To test this, future investigations will examine the faunal taphonomic histories of other rooms and occupational phases of Petsas House.

Currently, the closest parallels to the Petsas House well deposit are those from several well features at the site of Palaikastro in Crete. These well-preserved faunal assemblages similarly included complete dog and marten carcasses, young pig remains, as well as caprine, cattle, fish, and shell remains [67]. The dog remains were interpreted as potentially representing intentional, ritual deposits [68]. Many of these well deposits also appear to be related to household cleaning activities as they reflect remains of food mixed with construction debris, albeit over a broader time span from the Late Minoan IB to IIIA2 periods that overlap with the more temporally restricted Petsas House well deposit. Thus, around this time in the Aegean, it may have been a common practice to deposit complete and articulated dog carcasses in the bottom of wells that were also used as general refuse repositories. This may reflect wider cultural norms of intentional dog deposition, possibly for mixed ritual and practical purposes. These examples may also reflect multiple accidental deaths of free-roaming dogs, martens, and pests in hazardous open well features that were later filled.

### The faunal signatures of Mycenaean household subsistence

When combined with the taphonomic evidence, the detailed results of the livestock populations and body parts provided new insights into household access to food resources in different seasons at Mycenae. The well evidence suggests that Petsas House residents were at least partially provisioned with locally managed livestock resources, particularly pork. The evidence of young pig and caprine culling patterns from the middle part of the well fits a livestock management strategy aimed to maximize herd security, and thus, likely indicates more direct access to local animal resources [69]. Also, in the middle part of the deposit, body parts reflect higher proportions of the less desirable head and axial parts of livestock that likely indicate local butchery (e.g. [70]). This suggests access to herds through direct channels.

The low survivorship levels of pigs throughout the deposit support a scenario of direct local management, as pigs can give birth to large litters more than once a year and quickly grow to reach an optimal size for household meat production [71]. The Petsas House well deposits with a considerable abundance of young pig remains likely reflect locally produced meals, as pigs are an ideal choice for the small-scale independent provisioning of a household [72, 73]. As Mycenaean texts in Linear B script largely exclude accounts of pig rearing stock, management likely occurred in extra-palatial locales [1]. The evidence from the well suggests that Petsas House was likely one such locale.

Thus, household meals at Mycenae appear to have commonly included locally-reared pigs in the LH IIIA2 period. This confirms the recent finding that pigs played a larger, more integral role in the diets of Mycenaeans, and other Bronze Age Mediterranean cultures than previously suggested by the ancient texts [74, 75]. Still, pigs were likely used in more symbolic practices evidenced by the sacrificial burning of pig carcass parts at Methena and possibly Eleusis [28, 65]. We stress that only through more context-based studies of the taphonomic signatures of mundane animal use and disposal will we be able to reliably determine which types of carcass treatments evidence the special treatment of animals in ritual practice during the Late Helladic period. Otherwise, researchers will continue to rely on behavioral assumptions based on evidence from later classical period sites in Greece.

It is also of note that the variable intra-contextual aging results may reflect different seasons of caprine herd management. The gradual decrease in caprine survivorship across samples as the well was filled, or across clusters E to C, may reflect different goals of caprine rearing and culling at specific snapshots in time. This may point to an optimal meat production strategy, which typically targets more young male individuals for culling shortly after birth to maximize the survival of a manageable herd [72, 76]. The remaining herd would be gradually culled as needed given the wider economic demands of the community [77]. From the lower to the middle parts of the deposit, it appears that a greater proportion of the young population was killed off, leading up to a major cull of very young juveniles (cluster C), possibly during a lambing season. This was followed by a return to higher survivorship levels and culling of older animals. These shifts may have been impacted or inverted by the movement of the secondary and tertiary refuse. They also may reflect changing access to herds managed in different ways, as previous isotopic evidence suggested that the Petsas House caprines came from a mix of locales [8]. Future detailed demographic analysis of the caprine remains will test these hypotheses.

The aggregation of rubbish from various local meals likely also produced a heterogeneous pattern of livestock survivorship within the well deposit. The patterns differ from those expected for cases of catastrophic attrition [78]. Furthermore, we expect that kill-off goals to supply either an exceptionally large feast or smaller repeated ritual events would reflect more homogeneous survivorship levels throughout the deposit. For example, the burned sacrifices at Pylos appear to have required the repeated slaughter of numerous, mostly adult cattle [64]. Moreover, the faunal record of animal sacrifice at the site of Lykaion reflects a largely homogeneous record of burned, likely sacrificed remains of mostly upper hindlimb and tail parts of young adult caprines as practices were repeated over a long time, from the Late Helladic to the Late Classical periods [79, 80]. At Petsas House, the faunal composition of the well deposit varied more substantially in species abundance, livestock body part representation, and survivorship as well as in the taphonomic damage represented by depth.

Alternatively, our comparison of the samples of food refuse from middle and upper parts of the well suggests seasonal changes in the household diet. More abundant edible shells point to a narrow seasonal picture of refuse formation for clusters A–C, as the condition of *Arca noa* shells is best for consumption from late spring to early summer [81]. This seasonal signal overlaps with the evidence for the substantial kill-off of very young caprines and pigs in cluster C. Together, this may suggest that much of the upper part of the sample reflects meals from a late spring season that began with lambing and culminated in shellfish collecting.

Additionally, the higher proportions of cattle, dog, and hare remains with meaty limb parts of older livestock animals in the upper-most deposits represent a substantial contrast in the subsistence evidence from the middle deposits below. Many of these represent less optimal resources as dogs (with cut marks) often have more value alive for their social roles, hare meat is exceptionally lean, and shellfish have higher procurement costs given the substantial distance between Petsas House and the sea. While use of these types of resources may indicate greater subsistence stress, the upper-most deposit samples also contain higher proportions of cattle remains and limb parts of caprines and pigs—more marketable meaty animal resources that were more likely to have been accounted for by palatial authorities. Together, the mixed dietary picture from this part of the deposit fits expectations for access to some supplementary indirect animal resources provisioned through supply and redistribution either from the palace or through organized exchanges among craft and food producers [69, 82].

Thus, there appears to be more contrasting evidence of local and external subsistence provisions in the middle and upper parts of the well deposit. The sub-assemblage refuse differences may also reflect varied animal use and deposition in rooms with different functions. The complex may also have housed residents with different economic means and unequal access to animal resources. Further reconstruction of the associations among materials recovered from different parts of the well and different rooms of the house will test these hypotheses.

Overall, access to external provisioning systems, involving the palace, trade partners or possible markets, appears to have afforded the residents of Petsas House a high degree of subsistence resiliency. This reflects a more complex subsistence strategy for household risk minimization than would be possible through local agricultural diversification alone.

## Conclusion

Multiple lines of faunal evidence suggest that pre- and post-depositional processes had a disparate impact on the histories of refuse recovered from different parts of the well feature. The well assemblage provides a more context-specific view of household cleaning practice and provisioning than previously available at Mycenae.

Further consideration is needed of the different types of animal resources within the deposit in terms of the industrial and residential functions of the household. The faunal deposits appear to reflect a complex economic system that supported residents with food as they supplied the wider craft economy of the site and region, namely through the production of ceramic vessels [11]. While the well held evidence of different types of provisioning, likely through food and craft exchanges, further interpretation requires more detailed study of the butchery patterns and associated artifacts.

Although many pre-depositional activities with animals remain difficult to unravel with the zooarchaeological evidence alone (i.e. the dog carcasses at the bottom), the faunal depositional history of the well serves as an important starting place to disentangle the behaviors behind complex Mycenaean deposits. Through the application of faunal contextual taphonomy, our analysis defined sub-assemblages of possible carnivore interments, the contents of meals, and evidence of cleaning events. Our intra-context results also elucidated a faunal shift that may reflect times when the household received greater support from external sources, such as palatial authorities or trade partners who may have paid for ceramic vessels with food. The shifts may also reflect periods of subsistence stress around the time of the destruction event, differing access to resources among the Petsas House residents, or changing availability of food and completed vessels in different seasons.

These findings also demonstrate how previous faunal studies that lacked comparative, taphonomic analysis do not provide adequate resolution for understanding animal management and provisioning in terms of how animals were used at complex Mycenaean sites. Further taphonomic comparisons within and between contexts with statistical methods are required to better understand how interactions with animals impacted the resiliency of Mycenaean subsistence strategies in the face of shifting politics, changing trade systems, and unpredictable natural disasters of the Late Bronze Age.

## Supporting information

S1 File NISP taxonomic samples and sample sizes per level with the percentages of levels sampled. (XLSX)

S2 File Diversity t-test results. (XLSX)

S3 File Species abundance test results. (XLSX)

S4 File Data and results for the test of burned vs. unburned bones of medium-sized ungulates. (XLSX)

S5 File Data and results for the test of cut vs. uncut bones of medium-sized ungulates. (XLSX)

S6 File Data and results for the test of bones from medium-sized ungulates with green vs. dry breaks. (XLSX)

S7 File Data and results for the test of very young vs. older medium-sized ungulate remains. (XLSX)

S8 File Data and results for the test of mostly complete vs. incomplete bones of medium-sized ungulates. (XLSX)

S9 File Data and results for the test of weathered vs. not weathered bones of medium-sized ungulates. (XLSX)

S10 File Data and results for the test of gnawed vs. not gnawed bones of medium-sized ungulates. (XLSX)

S11 File Data and results for the test of smaller (< 2 cm) vs. larger (> 2 cm) bone specimens of medium-sized ungulates. (XLSX)

S12 File Bone fusion aging data and results by assemblage cluster. (XLSX)

S13 File Pig anatomical part analysis: Data and results. (XLSX)

S14 File Caprine anatomical part analysis: Data and results. (XLSX)

S15 File Dog anatomical part analysis: Data and results. (XLSX)

S16 File Caprine bone density test: Data and results. (XLSX)
